# A Systematical Study on Bands and Defects of CsBX_3_ (B = Pb, Sn, Ge, X = Cl, Br, I) Perovskite Based on First Principles

**DOI:** 10.3390/molecules29112479

**Published:** 2024-05-24

**Authors:** Chunqian Zhang, Hao Wang, Wenqi Huang, Yuhua Zuo, Jin Cheng

**Affiliations:** 1 Beijing Key Laboratory for Sensor, Beijing Information Science and Technology University, Beijing 100101, China; 2 School of Applied Science, Beijing Information Science and Technology University, Beijing 100101, China; 3 State Key Laboratory on Integrated Optoelectronics, Institute of Semiconductors, Chinese Academy of Sciences, Beijing 100083, China; yhzuo@semi.ac.cn

**Keywords:** inorganic lead-free perovskite, defects, first principles calculations

## Abstract

Metal halide perovskites have attracted considerable attention as novel optoelectronic materials for their excellent optical and electrical properties. Inorganic perovskites (CsPbX_3_, X = Cl, Br, I) are now viable alternative candidates for third-generation photovoltaic technology because of their high photoelectric conversion efficiency, high carrier mobility, good defect tolerance, simple preparation method and many other advantages. However, the toxicity of lead is problematic for practical implementation. Thus, the fabrication of lead-free perovskite materials and devices has been actively conducted. In this work, the energy band and photoelectric properties of inorganic perovskites CsBX_3_ (B = Pb, Sn, Ge, X = Cl, Br, I) have been investigated with the first principles calculation, and the possible defect energy levels and their formation energies in different components, in particular, have been systematically studied. The advantages and disadvantages of Sn and Ge as replacement elements for Pb have been demonstrated from the perspective of defects. This study provides an important basis for the study of the properties and applications of lead-free perovskites.

## 1. Introduction

Perovskites are very important photoelectric materials. Due to their characteristics of high chromatography purity, suitable tunable band gap, high photoluminescence quantum yield and high carrier mobility [1,2,3,4,5], they have been intensively studied in the application of high-efficiency light-emitting diodes, solar cells, detectors and other fields [6,7,8,9,10]. Perovskite was first applied to solar cells in 2009 with an initial power conversion efficiency of only 3.8% [11], and this number has exceeded 25% after a development of more than ten years [12,13]. However, as a heavy metal ion, Pb^2+^ may harm human health and cause soil pollution [6], which brings toxicity problems that cannot be ignored, and various countries have introduced restrictions on lead. Therefore, lead-free perovskite materials with excellent characteristics have become an important direction for researchers.

ABX_3_ structure is the most typical of perovskite compositions, among which the stability of pure inorganic perovskites is more prominent, and the most common one is CsPbX_3_ (X = Cl, Br, I). Replacing Pb^2+^ in CsPbX_3_ with non-toxic metal cations with similar ionic radii and properties is a good method to solve the toxicity of perovskite materials. Wu et al. reported Ge-based perovskite nanocrystals synthesized by the solution method for the first time in 2018 [14]. They also investigated the electron beam-induced transformations of CsGeI_3_ and found a distinctive transformation path compared to heavier Pb atoms in lead halide perovskite NCs. To improve the poor stability of lead-free perovskite, Kang et al. highlight the significant effect of Sn-II precursors used in the synthesis of the resultant CsSnX_3_ NCs. Stable CsSnX_3_ NCs can be obtained with the use of antioxidative SnC_2_O_4_ as the Sn-II precursor for the strong antioxidative ability of the oxalate ion [15]. In addition to lead-free perovskite materials, their related applications have also received attention from scientists. High-quality Cs_3_Bi_2_I_9_ perovskite nanosheets have been prepared and used in solar cells [16], and it has been proved that Bi-based perovskites have more similar properties with Pb-based ones and also have high quantum yields compared to other nontoxic ions [17,18]. Sun et al. developed new fullerene derivatives as electron transporter layers, and found that the chemical structures of the regioisomers not only affect their energy levels, but also lead to significant differences in their molecular packings and interfacial contacts [19]. They reported an efficiency of 14.30%, representing one of the best performances of Sn-based perovskite solar cells. Lead-free perovskites have also shown great promise for the photochemical conversion of CO_2_ [20,21,22]. However, the fast carrier recombination rates and inadequate adsorption/activation for CO_2_ molecules have seriously restricted their practical application. Qian et al. developed an innovative Cs_2_CuBr_4_/TiO_2_ photocatalyst by embedding Cs_2_CuBr_4_ PQDs in a mesoporous TiO_2_ matrix, which delivered the CO_2_ reduction activity to 3.1 and 16.0 times higher than those achieved by pure Cs_2_CuBr_4_ and TiO_2_, respectively [21].

Better defect property is one of the most important advantages of Pb-based perovskites compared to other optoelectronic materials, which is of great significance to the material properties as well as the performance of optoelectronic devices. Generally, in optoelectronic devices, deep-energy-level defects mainly act as recombination centers, shorten the lifetime of minority carriers and reduce the device efficiency, while shallow-energy-level defects are mainly donor or acceptor, which mainly provide carriers and induce the yield of the device. In addition to the location of the energy level, the formation energy is also an important influence factor for the defect concentration. For Pb-based perovskite, such as MAPbI_3_, most of the defects (I_i_, MA_Pb_, V_MA_, V_Pb_, MA_i_, Pb_MA_, V_I_ and MA_I_) are located above the conduction band minimum (CBM) or below the valence band maximum (VBM), forming shallow defect energy levels, and meanwhile have relatively low formation energies, resulting in higher concentrations. However, for a few other defects (I_MA_, I_Pb_, Pb_i_ and Pb_I_), they are located in the middle of the forbidden band and form deep defect energy levels. Fortunately, their relatively higher formation energies ensure lower concentrations and less impact [23,24]. This is the reason why Pb-based perovskites have better defect tolerance [23,25]. It is hoped that lead-free perovskites will have similar properties so as to minimize the negative effects of defects. 

CsSnI_3_ is an early proposed lead-free perovskite material, and there have been many reports on the research of its defects. In 2012, Chung et al. made a computational study of several point defects in CsSnI_3_ and found that the Sn vacancies mainly act as p-type carrier sources and have lower formation energies. Thermal-activated Sn vacancies improve conductivity and PL intensity of CsSnI_3_ [26]. Xu et al. further investigated the possible defects in CsSnI_3_ to verify the intrinsic defect energy level distribution in CsSnI_3_ [27]. It was shown that all the transfer energy levels of the acceptor defects are below the VBM and will all ionize once formed, increasing the carrier concentration. Among the donor defects, Sn_I_ is the only deep-energy-level defect that will exist as a recombination center of carriers, whose density can be reduced by increasing the concentration of Sn in the precursor. The intrinsic defects of MASn_x_Pb_1−x_I_3_ were also studied with first principles calculations [28]. It was found that three defects (I_i_, V_Sn_, and V_Pb_) have a large impact on the carrier lifetime. I_i_ introduces a deep defect state in the forbidden band and leads to a significant decrease in carrier lifetime and PL intensity, which fortunately can be effectively avoided by reducing the anion content. Neither V_Sn_ nor V_Pb_ produce defect energy in the band gap; however, they can cause changes in the energy dispersions of VBM and CBM, which in turn change the carrier mobility and lifetime. Although the previous reports on the defect properties of lead-free perovskite have important guiding significance, most of them only focus on several single materials, and the results are not comprehensive enough. Here, we have specifically conducted a systematic and comprehensive study of all the possible point defects in Sn- and Ge-based perovskites CsBX_3_ (B = Pb, Sn, Ge, X = Cl, Br, I), the two optimally desired replacement elements for Pb, and studied the distribution of defect energy levels for different metal and halogen elements. 

In this paper, CsBX_3_ (B = Pb, Sn, Ge, X = Cl, Br, I) perovskites are investigated by first principles calculations. The electronic properties of perovskites, including energy band structure, density of states (DOS), and absorption coefficients, are firstly studied. On this basis, the energy level and distribution of all possible point defects are explored in depth to analyze the defect tolerance of the material. Finally, the DOS distribution of the defect lattice is calculated, which can explain the influence of defect location on carrier concentration well. This study work not only reveals the mechanisms of the defects’ existence and their effect on the material optoelectronic properties, but also provides experience on the experimental preparation of different materials, and the prediction of the device performance with different materials.

## 2. Models and Calculation Methods

**Figure 1:**
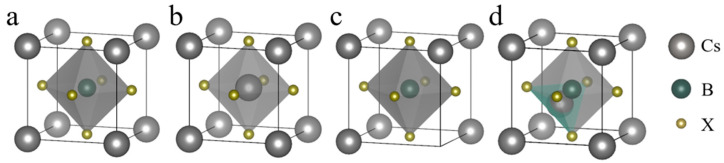
CsBX_3_ perovskite ideal and defective crystals. (**a**) Ideal cell. (**b**) Cs_B_ (Cs substitution B defect). (**c**) V_Cs_ (Cs vacancy defects). (**d**) Cs_i_ (Cs interstitial defects). Each type of defect shows only one type of cell.

At room temperature, CsBX_3_ perovskite is cubic crystal and the space group is Pm-3m. The unit cell contains five atoms in a single formula unit, and the positions with fractional coordinates (0.00, 0.00, 0.00), (0.50, 0.50, 0.50), and (0.00, 0.50, 0.50) are occupied by Cs, B, and X atoms, respectively. B^2+^ and its coordinated X^−^ form an octahedral, B^2+^ is the center of the octahedral structure, and X^−^ is located at the top corner of the octahedron. A unit cell is shown in Figure 1a. There are three types of point defects in the cell, namely substitution, vacancy and gap, and Figure 1b–d show one cell structure for each type of defect, respectively.

Here, the valence electronic configurations of the atoms are as follows: Cs: 5s^2^5p^6^6s^1^, Pb: 3d^6^4s^2^, Sn: 5s^2^5p^2^, Ge: 4s^2^4p^2^, Cl: 3s^2^3p^5^, Br: 4s^2^4p^5^, I: 5s^2^5p^5^. The Perdew–Burke–Ernzerhof functional for solids (PBE) within the generalized gradient approximation (GGA) was used to describe the exchange correlation interaction between the valence electrons. As the major computational parameters, the plane-wave cutoff energy was set to 405 eV, and the Monkhorst–Pack special k points were set to (6 × 6 × 6) for structural relaxations and electronic structure calculations. To obtain more reliable band gaps, we also performed electronic structure calculations using the Heyd–Scuseria–Ernzerhof (HSE06) hybrid functional, in which we replaced 25% of the PBE exchange functional with the exact Hartree–Fock exchange functional, providing bulk band gaps in good agreement with the experiment. Subsequently, the light absorption capacity of the material was also calculated.

To study the distribution of point defects in perovskite, we calculated the energy level locations and formation energies of all possible defects formed in CsBX_3_, including vacancy, substitution and interstitial. For a defect α with charged state q, the formation energy ΔH (α, q) is calculated as shown below:

ΔH (α, q) = E (α, q) − E (host) + ∑*_i_n_i_μ_i_* + *q* [E_VBM_ (host) + E_f_ + ∆V] (1)

where E (α, q) is the energy of the supercell with defect α in ionization state *q*, E (host) is the energy of the supercell without defect, *n_i_* is the number of atoms added or lost from defect α, *μ_i_* is the chemical potential of the corresponding element, E_VBM_ (host) is the energy at the VBM of the supercell without defect, and E_f_ is the Fermi energy relative to E_VBM_ (host), i.e., E_f_ = 0 at the position of E_VBM_ (host), ∆V = V (α, q, R) − V (host, R), where R is the farthest position from the defect. Here, the formation energy of the defect is calculated with PBE generalization. However, the PBE calculation underestimates the formation energy per electron ∆CBM when there are electrons occupying the host energy level (when there are holes occupying the host energy level, the PBE calculation underestimates the formation energy per hole ΔVBM) [29], so we added the difference in the conduction (valence) band edge energy generated by HSE06 to correct the defect formation energy calculated by PBE.

## 3. Discussion

### 3.1. ABX_3_ Crystal Structure

As a preliminary test of the formability of perovskite structures, the Goldschmidt tolerance factor *t_G_* was first analyzed, and the calculation formula is shown in Formula (2):

*t_G_* = (*r_A_* + *r_X_*)/(2^1/2^ (*r_B_* + *r_X_*)) (2)

where *r_A_* represents the ionic radius of A, *r_B_* represents the ionic radius of B, and *r_X_* represents the ionic radius of X. When the calculated result is in the range of 0.8~1.0, it can support the formation of perovskite structure in a stable phase [30]. After the cell is established, the relaxation calculation is carried out to optimize the structure. Table 1 shows the lattice parameters and tolerance factors of the perovskite materials studied thereinafter.

It can be seen from the table that the lattice constants of the perovskites with the same B element gradually increase when the X elements are Cl, Br and I in order, the lattice constants of the perovskites with the same X element also gradually increase when the B elements are Ge, Sn, and Pb in order, which is consistent with the change in ionic radii of the constituent elements. The tolerance factors of all nine materials are in the range of 0.8~1.0, which ensures the formation of the perovskite structure.

### 3.2. Band Strucure

The energy band is the fundamental factor that determines the optoelectronic properties of semiconductor materials. We calculated the energy bands of CsBX_3_ (B = Pb, Sn, Ge, X = Cl, Br, I) after relaxation, and the PBE and HSE06 software packages were used, respectively, to explore the details of the electronic structure. Since the ABX_3_ perovskite belongs to the Pm-3m space group, we selected the high symmetry point paths in the Brillouin zone as X (0.50, 0.00, 0.00)-R (0.50, 0.50, 0.50)-M (0.50, 0.50, 0.00)-T (0.00, 0.00, 0.00)-R (0.50, 0.50, 0.50). The band gaps of the materials are summarized in Table 2. The band gap obtained from the PBE generalized function calculation is severely underestimated compared to the experimental value, which is consistent with the previous theoretical results [30]. This underestimation of the band gap becomes more severe as the halogen atoms become lighter when changing from I to Cl due to weaker relativistic effects [30]. The HSE06 hybridization generalization significantly improves this defect and yields a band gap closer to the experimental value due to a fraction of screened Hartree–Fock exchange included in HSE06, which improves the discontinuity in the Kohn–Sham potential derivative for integer numbers of electrons.

**Figure 2:**
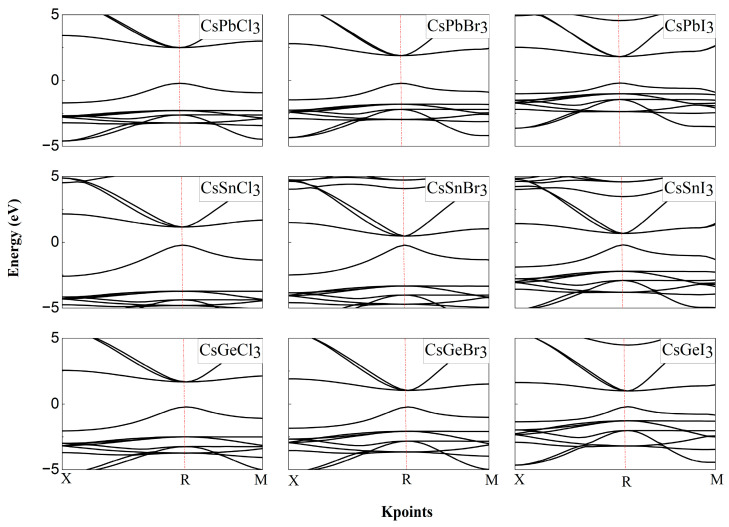
CsBX_3_ band structure obtained by HSE06 functional calculation.

The energy band structures obtained by the HSE06 hybridization generalization calculation are given in Figure 2. Sn-, Ge- and Pb-based perovskites have similar energy band structures. They all have direct band gaps, the inverse spatial coordinates of the CBM and VBM are located at the R (0.50, 0.50, 0.50), and the band formation and order are similar. This is because the chemical composition and the backbone structure formed by B and X are similar. The direct band gap structure makes it easier for the carrier transition from the VBM to the CBM generated by photons without the assistance of phonons, and facilitates the generation of photogenerated carriers, which makes Sn- and Ge-based perovskites equally promising for the application of high-efficiency optoelectronic devices. The band gap decreases with increasing halogen ion radius from Cl to I, which has been demonstrated in previous work, and provides a classical way to regulate the band gap by controlling the halogen composition [40,41]. This may be because the longer B-X bonds weaken the interaction between B and X orbitals, leading to a narrowing of the band gap while causing lattice expansion [42,43]. The effect of B elements on the band gap is significant and more complex, which cannot be explained well by bond length alone and needs to be further investigated.

**Figure 3:**
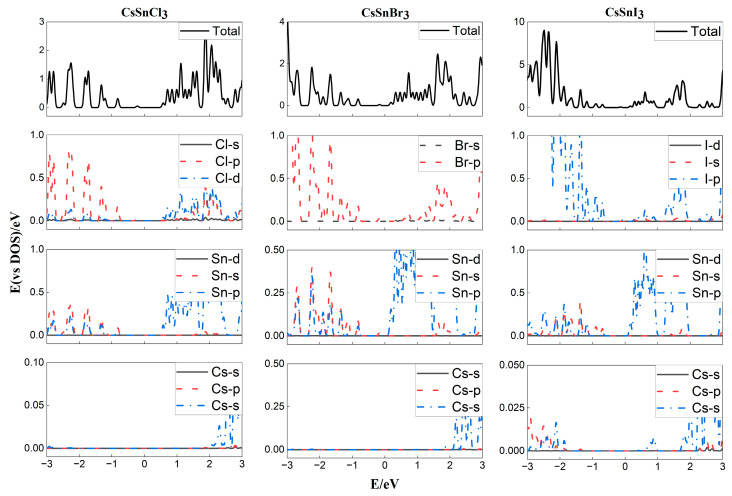
DOS of CsSnX_3_ perovskites.

The DOS distribution is another important parameter for studying the properties of optoelectronic materials, as it reveals the contribution of material composition elements to the energy bands and distribution of charge carriers. Here, the DOS of the above CsBX_3_ is calculated. The results are shown in Figure 3. It can be observed that the VBM mainly originates from the p orbitals of X and a small amount of s orbitals of B, while the CBM is mainly composed of the p orbitals of B and a small amount of p and d orbitals of X. Their overlap indicates significant hybridization between each other. That is, the band gap is mainly determined by the B and X elements, and the contribution of the A (Cs) is negligible, which is why changing the elements in the A-site has little effect on the band gap of the material [1]. This is consistent with the conclusion in Figure 2.

A high absorption coefficient is critical for efficient photoelectric conversion of the material. The absorption coefficient of perovskite is derived from the real and imaginary parts of the dielectric function together. It is calculated as α(ω) = 2^1/2^e/ħc [(ε_1_^2^+ε_2_^2^)^1/2^ − ε_1_]^1/2^α, where ε_1_(ω) and ε_2_(ω) are the real and imaginary parts of the dielectric function, respectively, and ω is the frequency of the photon [44].

**Figure 4:**
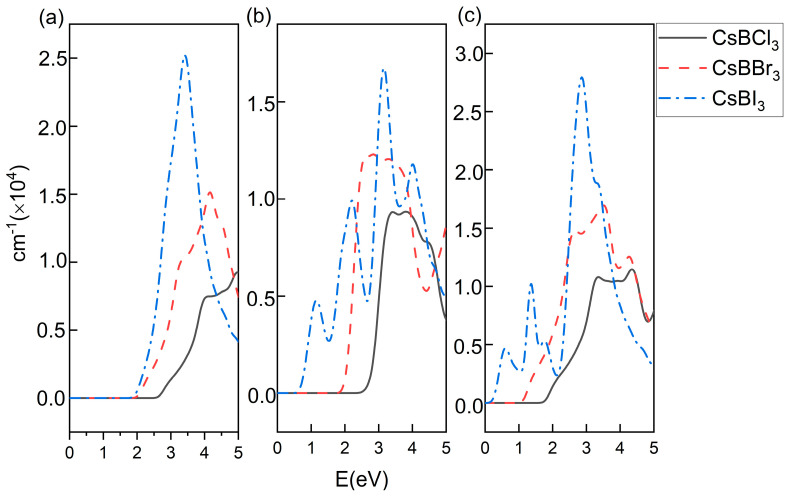
ABX_3_ perovskite (**a**) B = Pb, (**b**) B = Sn, (**c**) B = Ge absorption spectrum.

The light absorption spectra of the nine materials are given in Figure 4. It can be found that when B changes from Pb to Ge, the absorption edge gradually produces a red shift, and for the same B-site element, when X is Cl, Br and I elements, the absorption curve also shows a more obvious red shift in turn, which corresponds to the size of the band gap. They all have good absorption ability in the visible range, while the Ge- and Sn-based perovskites also have some absorption ability in the infrared region, giving them greater advantages in expanding the absorption scope. Secondly, the absorption peaks of the same B-element materials become larger in the order of X for Cl, Br, and I, which is consistent with the variation in the absorption scope.

### 3.3. Defect Property

**Figure 5:**
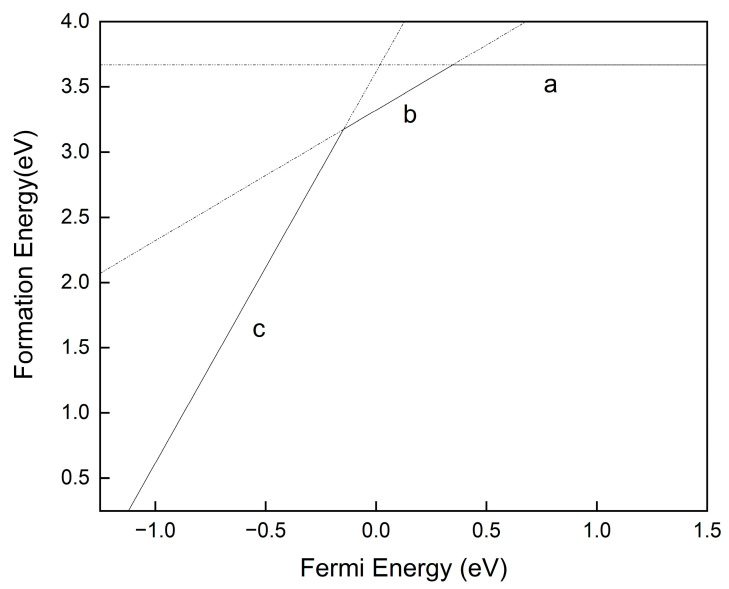
Sn_I_ defect energy levels in CsSnI_3_. Section a is for electrically neutral state, section b is for +1 charged state and section c is for +3 charged state.

**Figure 6:**
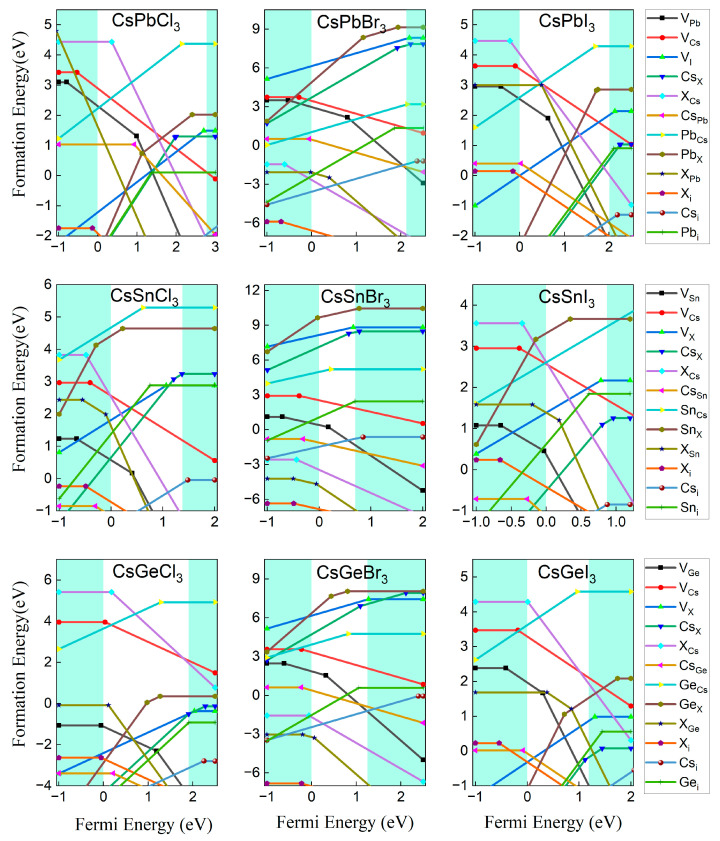
The calculated defect formation energy as a function of the Fermi energy. According to Equation (1), the slope of the function indicates the charge state q of the defect, and the Fermi energy at the turning point gives the transition energy level. The shaded areas on the left and right sides of the figure indicate the valence band below the VBM and the conduction band above the CBM, respectively.

The properties of defects in absorbers, especially point defects, play a critical role in determining the electron–hole diffusion length and V_oc_ of a solar cell. Defects that create deep levels usually act as Shockley–Read–Hall nonradiative recombination centers and are responsible for short minority carrier lifetime and, thus, low V_oc_. In contrast, defects with energy levels above the CBM or below the VBM can increase the carrier concentration and improve the efficiency of the device. The defect formation energy is a very important reference to understand the defects’ distribution in semiconductor materials. The higher the formation energy, the lower the chance of defect formation, and also its concentration in the material. Here, we take the calculation result of Sn_I_ (Sn substitution of I) defect in CsSnI_3_ as an example, to describe the significance of the defect formation energy curve calculated by first principles. Sn_I_ may exist in three charged states, i.e., electrically neutral, with +1 charge, and with +3 charge. The formation energy in each charged state is calculated in accordance with Equation (1), and the results are shown in Figure 5. The formation energy of neutral defects does not vary with the E_f_, while that of charged defects (Sn_I_^1+^ and Sn_I_^3+^) increases with increasing E_f_ (the slope of the curve is equal to the charge number of the defect), which also applies to those negatively charged acceptor defects. Only the charged state with the lowest formation energy can exist at any Fermi energy, so the actual formation energy curve is composed of section a for electrically neutral state, section b for +1 charged state and section c for +3 charged state. According to Equation (1), the formation energy versus Fermi energy for all possible defects in ABX_3_ perovskites were predicted, including V_Cs_ (Cs vacancy), V_B_, V_X_, Cs_B_ (Cs substitution of B), B_Cs_, Cs_X_, X_Cs_, B_X_, X_B_, Cs_i_ (Cs gap), B_i_ and X_i_, and the calculation results are shown in Figure 6.

In order to better observe the changes in formation energy, we have listed the two maximum and minimum formation energies of each donor and acceptor defect for nine materials, as shown in Table 3, Table 4, Table 5 and Table 6. It can be clearly seen that the formation energy of defect is much more influenced by X element than by B element. Because the generated defects can have a significant impact on the material, we focus more on the donor and acceptor defects with lower formation energies. In most materials, Cs_i_ and B_i_ are the donor defects with the lowest formation energy, but when X is I, Cs_X_ replaces the position of B_i_. This is easy to understand, because the Cs ions do not participate in the skeletal structure that constitutes the perovskite, but rather fill the octahedral structure composed of metal and halogen ions, making them subject to the least binding forces and more likely to form defects, which agrees with the previous conclusions [23]. Due to the larger ionic radius of I, it is more easily replaced by Cs, making the formation energy of Cs_I_ relatively low. For the acceptor defects, Cs_B_ and X_i_ have relatively low formation energies for CsPbCl_3_ and CsPbI_3_, while X_B_ becomes more easily formed in CsPbBr_3_. It is believed that the difference in the compounds containing Br ions is caused by multiple factors. Due to the low local symmetry of the X site, the 5p orbital of B splits forms a shallow energy level near the CBM, but also a deep energy level in the forbidden band [27]. Although the three halogen atoms have similar electronic structures and also orbital couplings with B ions, the coupling strength of Cl with B is stronger due to a smaller atomic radius, while those of Br and I will gradually weaken as the atomic radius increases. On the other hand, as discussed above, the radius of the ion plays a key role in the stability of the compound structure and the formation of defects. Cl ions with smaller radii do not have an advantage in forming stable compounds, while Br and I can form more stable compounds with fewer defects as the ion radius increases. The balance and contest between these two factors make it easier for the intermediate Br to escape or to be replaced by other ions, forming vacancy or substitution defects, which results in the conclusion we finally obtained. This is consistent with the research results of W. Swift et al., that Br_i_ in CsPbBr_3_ is a deep-level defect with a high concentration [45].

**Figure 7:**
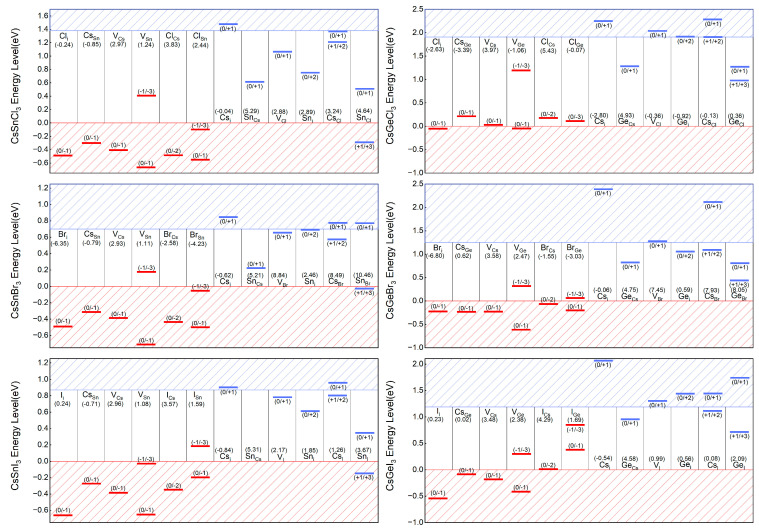
The calculated transition energy levels for various intrinsic defects of CsBX_3_ (B = Sn, Ge. X = Cl, Br, I). The regions under VBM and above CBM are in the slash area. The acceptor and donor defect levels are indicated by red and blue lines, respectively, and the numbers in parentheses above represent the change in defect charging. The number in parentheses below each defect name represents the formation energy for each defect when electrically neutral.

Ionization is an important parameter for determining the contribution of defect in carrier concentration, which is mainly influenced by their transition energy levels. The transition energy level ε_α_(q/q’) is defined as the Fermi energy level when defect α with two different charge states q and q’ have the same formation energy [46], and they are located at the turning points in the formation energy diagram, where the defects can release electrons or holes and change their charge state from q to q’. For example, the Fermi energy level E_f_ = −0.66 eV corresponds to the transition point of the I_i_ from the electroneutral to the −1-valence state in the CsSnI_3_ system, so that its (0/−1) transition energy level is located at 0.66 eV below the VBM. Based on this approach, the positions of the transition energy levels of defects in Sn- and Ge-based perovskites are plotted in Figure 7. The formation energy and the energy level are the two main factors determining the distribution of a defect in the material. The acceptor defect with a transition energy level below the VBM or the donor defect with a transfer energy level above the CBM will ionize once formed, increasing the concentration of carriers. However, if the transition energy level of the defect lies in the forbidden band, the defect will then act as a carrier trap and recombination center for the photogenerated electron–hole pair, decreasing the carrier concentration.

Many previous studies have pointed out that the good defect property is one of the important reasons why Pb-based perovskites are sought after by scholars for optoelectronic device applications [23,47,48]. Most of the defects in Pb-based perovskites have shallow energy levels, and the formation energy of the very few with deep energy levels is relatively higher, meaning they are difficult to form and they affect the performance of the material vary little [23,49].

Fortunately, the defect properties of Sn-based perovskites are similar to those of Pb-based ones. Almost all of the acceptor defect transition energy levels in CsSnX_3_ are below the VBM, which means they will all ionize once formed. This conclusion is similar to that of a previous study [27]. Each ionized defect produces the same number of carriers as the amount of charge in its ionized state. Therefore, a high concentration of shallow-energy-level defects causes a higher concentration of carriers and further conductivity of the material. Although there are some deep-energy-level donor and acceptor defects, such as Sn_Cs_, V_Cl_, Cs_Cl_, Sn_Cl_ in CsSnCl_3_, their formation energies are relatively high, hindering the mass formation of recombination centers. At the same time, it is important to note that defects cannot capture two electrons or holes at the same time [50,51], which means that transfer energy levels like (−1/+1), (−2/0), and (0/+2) that produce transitions of two or more charge states are actually not related to nonradiative recombination. Therefore, V_Sn_ and Sn_i_ are not actually nonradiative recombination centers.

In contrast, the performance of Ge-based perovskites is not so satisfactory. A number of deep-energy-level defects with low formation energies (Cs_Ge_, V_Ge_, Cl_Ge_ and Ge_Cl_ in CsGeCl_3_, Ge_i_ in CsGeBr_3_, and Cs_I_ in CsGeI_3_) provide conditions for the existence of carrier recombination centers. CsGeI_3_ and CsGeBr_3_ are significantly more tolerant to defects than CsGeCl_3_, but undesirable defects exist in both of them as well. The defect properties of Sn- and Ge-based halide perovskites explained the advantages of Sn as the substitute for Pb, and the rapid growth of Sn-based perovskite compared with Ge-based ones.

**Figure 8:**
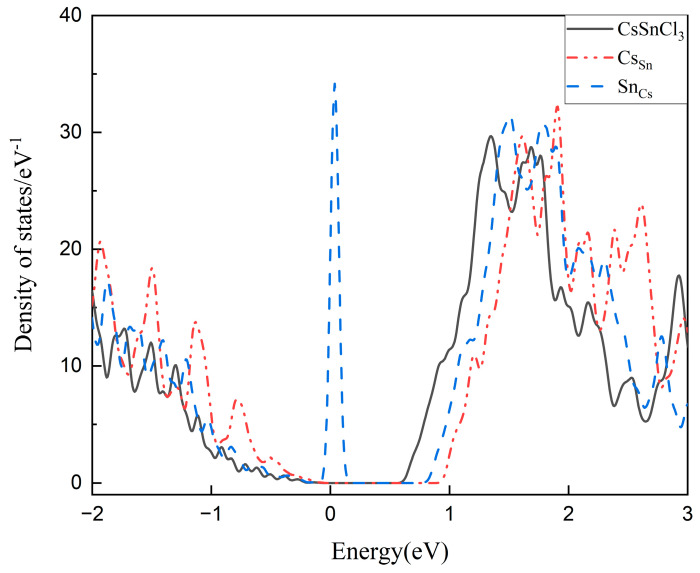
Total DOS of ideal CsSnCl_3_, with defect Cs_Sn_ and Sn_Cs_.

The DOS in crystals, including those of defects that are studied here, to investigate the influence of defect energy levels and formation energies on carrier contribution. Two defects (Cs_Sn_ and Sn_Cs_ in CsSnCl_3_) are studied as a special case, for they are typical ones with shallow and deep energy levels, respectively, and their effect on the carrier state density can specifically illustrate the influence of most defects on carrier concentration and mobility in crystals. The results are shown in Figure 8. It can be clearly observed that Cs_Sn_ caused complete ionization, which resulted in a higher DOS below the VBM, and increased the carrier concentration in the crystal, while for deep-energy-level defect Sn_Cs_, a recombination center was created in the middle of the forbidden band, which is consistent with our previous analysis.

## 4. Conclusions

The article presents a systematic and comprehensive study of the photoelectric properties of CsBX_3_ (B = Pb, Sn, Ge. X = Cl, Br, I.) perovskites, especially the properties of defect energy levels and formation energies, based on the first principle calculations. There is a significant effect of B and X elements on the band gap, while the effect of Cs elements on the band gap is small. The perovskites constructed with different B-site elements have good absorption ability in the visible range, while the absorption edges of the Ge- and Sn-based perovskites show different degrees of red-shift, which makes them also have some absorption in the infrared region and expands the wavelength range for material applications.

The defect energy levels and formation energies of all possible point defects in CsBX_3_ (B = Pb, Sn, Ge, X = Cl, Br, I) perovskite are systematically investigated. Sn-based perovskites have similar defect properties with Pb-based material, where most of the defects are near the CBM or the VBM, and form shallow-energy-level defects. For a few defects (Sn_Cs_, V_Cl_, Sn_Cs_, Cs_Cl_ and Sn_Cl_) existing in the middle of the forbidden band, high formation energies ensure their low concentration, which proves the mobility of Sn-based perovskite and makes it a promising lead-free material. In contrast, there are some defects with deep energy levels in Ge-based perovskites (Ge_Cl_ in CsGeCl_3_, Ge_i_ in CsGeBr_3_, and Cs_I_ in CsGeI_3_) with relatively lower formation energies, forming carrier capture centers and hindering their application in optoelectronic devices. These analysis results indicate that Sn has more advantages in defect tolerance when replacing Pb to become an efficient perovskite optoelectronic material. This is the main reason why Sn-based perovskite devices are superior to Ge-based ones. This work is of great significance for the study of lead-free perovskites and provides a basis for theoretical and experimental studies of lead-free perovskites and devices.

## Figures and Tables

**Table 1:** Lattice constants and tolerance factors of CsBX_3_ (B = Pb, Sn, Ge, X = Cl, Br, I) cell structure diagram.

Materials	CsPbCl_3_	CsPbBr_3_	CsPbI_3_	CsSnCl_3_	CsSnBr_3_	CsSnI_3_	CsGeCl_3_	CsGeBr_3_	CsGeI_3_
Lattice constants	5.73	5.99	6.42	5.55	5.79	6.21	5.43	5.64	5.98
Tolerance factor	0.82	0.81	0.81	0.90	0.89	0.88	0.97	0.96	0.94

**Table 2:** CsBX_3_ band gap (unit: eV) summary.

Materials	PBE	HSE06	HSE06 Value	Experimental Value
CsPbCl_3_	2.15	2.78	2.89 [31]	3.00 [32]
CsPbBr_3_	1.68	2.12	2.35 [31]	2.25 [33]
CsPbI_3_	1.45	2.01	2.02 [34]	1.73 [35]
CsSnCl_3_	0.94	1.38	1.50 [31]	2.90 [36]
CsSnBr_3_	0.39	0.70	1.05 [31]	1.75 [37]
CsSnI_3_	0.40	0.87	0.74 [31]	1.27 [37]
CsGeCl_3_	1.36	1.91	1.69 [31]	3.31 [38]
CsGeBr_3_	0.90	1.25	1.15 [31]	2.37 [38]
CsGeI_3_	0.71	1.19	1.55 [31]	1.51 [39]

**Table 3:** Two donor defects with maximum formation energies.

	X = Cl	X = Br	X = I
B = Pb	B_Cs_, B_X_	B_X_, V_X_	B_Cs_, B_X_
B = Sn	V_X_, B_Cs_	B_X_, V_X_	V_X_, B_X_
B = Ge	B_Cs_, B_X_	B_X_, Cs_X_	B_Cs_, B_X_

**Table 4:** Two donor defects with minimum formation energies.

	X = Cl	X = Br	X = I
B = Pb	B_i_, Cs_i_	B_i_, Cs_i_	B_i_, Cs_i_
B = Sn	B_i_, Cs_i_	B_i_, Cs_i_	Cs_X_, Cs_i_
B = Ge	B_i_, Cs_i_	B_i_, Cs_i_	Cs_X_, Cs_i_

**Table 5:** Two acceptor defects with maximum formation energies.

	X = Cl	X = Br	X = I
B = Pb	X_B_, X_Cs_	V_Cs_, V_B_	X_Cs_, V_Cs_
B = Sn	X_Cs_, V_Cs_	V_Cs_, V_B_	X_Cs_, V_Cs_
B = Ge	X_Cs_, V_Cs_	V_Cs_, V_B_	X_Cs_, V_Cs_

**Table 6:** Two acceptor defects with minimum formation energies.

	X = Cl	X = Br	X = I
B = Pb	Cs_B_, X_i_	X_B_, X_i_	Cs_B_, X_i_
B = Sn	X_i_, Cs_B_	X_B_, X_i_	X_i_, Cs_B_
B = Ge	X_i_, Cs_B_	X_B_, X_i_	X_i_, Cs_B_

## Data Availability

The data that support the findings of this study are available from the corresponding author upon reasonable request.

## References

[1] Mao X., Sun L., Wu T., Chu T.S., Deng W.Q., Han K.L. (2018). First-Principles Screening of All-Inorganic Lead-Free ABX_3_ Perovskites. J. Phys. Chem. C.

[2] Kshirsagar B., Jaykhedkar N., Jain K., Kishor S., Shah V., Ramaniah L.M., Tiwari S. (2021). Green CsSnX_3_ (X = Cl, Br, I)-Derived Quantum Dots for Photovoltaic Applications: First-Principles Investigations. J. Phys. Chem. C.

[3] Guo H., Yoon G.W., Li Z.J., Yun Y., Lee S.W., Seo Y.H., Jeon N.J., Han G.S., Jung H.S. (2024). In Situ Polymerization of Cross-Linked Perovskite-Polymer Composites for Highly Stable and Efficient Perovskite Solar Cells. Adv. Energy Mater..

[4] Ali L., Ahmad M., Shafiq M., Zeb T., Ahmad R., Maqbool M., Ahmad I., Jalali-Asadabadi S., Amin B. (2020). Theoretical studies of CsSnX_3_ (X= Cl, Br and I) for energy storage and hybrid solar cell applications. Mater. Today Commun..

[5] Milstein T.J., Kroupa D.M., Gamelin D.R. (2018). Picosecond quantum cutting generates photoluminescence quantum yields over 100% in ytterbium-doped CsPbCl_3_ nanocrystals. Nano Lett..

[6] Lin W., Hu X., Mo L., Jiang X., Xing X., Shui L., Priya S., Wang K., Zhou G. (2021). Progresses on Novel B-Site Perovskite Nanocrystals. Adv. Opt. Mater..

[7] Zhao Y., Ma F., Qu Z.H., Yu S.Q., Shen T., Deng H.X., Chu X.B., Peng X.X., Yuan Y.B., Zhang X.W. (2022). Inactive (PbI_2_)_2_RbCl stabilizes perovskite films for efficient solar cells. Science.

[8] Gao Y., Ren F.M., Sun D.R., Li S.B., Zheng G.H.J., Wang J.A., Raza H., Chen R., Wang H.X., Liu S.W. (2023). Elimination of unstable residual lead iodide near the buried interface for the stability improvement of perovskite solar cells. Energy Environ. Sci..

[9] Li J.H., Du P.P., Guo Q.X., Sun L., Shen Z.X., Zhu J.X., Dong C., Wang L., Zhang X., Li L.Y. (2023). Efficient all-thermally evaporated perovskite light-emitting diodes for active-matrix displays. Nat. Photonics.

[10] Zhou Y., Fei C.B., Uddin M.A., Zhao L., Ni Z.Y., Huang J.S. (2023). Self-powered perovskite photon-counting detectors. Nature.

[11] Kojima A., Teshima K., Shirai Y., Miyasaka T. (2009). Organometal Halide Perovskites as Visible-Light Sensitizers for Photovoltaic Cells. J. Am. Chem. Soc..

[12] Gao H., Xiao K., Lin R., Zhao S., Wang W., Dayneko S., Duan C., Ji C., Sun H., Bui A.D. (2024). Homogeneous crystallization and buried interface passivation for perovskite tandem solar modules. Science.

[13] Zhou J., Tan L., Liu Y., Li H., Liu X., Li M., Wang S., Zhang Y., Jiang C., Hua R. (2024). Highly efficient and stable perovskite solar cells via a multifunctional hole transporting material. Joule.

[14] Wu X., Song W., Li Q., Zhao X., He D., Quan Z. (2018). Synthesis of Lead-free CsGeI3 Perovskite Colloidal Nanocrystals and Electron Beam-induced Transformations. Chem.—Asian J..

[15] Kang C., Rao H., Fang Y., Zeng J., Pan Z., Zhong X. (2021). Antioxidative Stannous Oxalate Derived Lead-Free Stable CsSnX3 (X=Cl, Br, and I) Perovskite Nanocrystals. Angew. Chem. Int. Ed..

[16] Bai F., Hu Y.H., Hu Y.Q., Qiu T., Miao X.L., Zhang S.F. (2018). Lead-free, air-stable ultrathin Cs3Bi2I9 perovskite nanosheets for solar cells. Sol. Energy Mater. Sol. Cells.

[17] Zhao S.Y., Cai W.S., Wang H.X., Zang Z.G., Chen J.Z. (2021). All-Inorganic Lead-Free Perovskite(-Like) Single Crystals: Synthesis, Properties, and Applications. Small Methods.

[18] Tress W., Marinova N., Moehl T., Zakeeruddin S.M., Nazeeruddin M.K., Gratzel M. (2015). Understanding the rate-dependent J-V hysteresis, slow time component, and aging in CH_3_NH_3_PbI_3_ perovskite solar cells: The role of a compensated electric field. Energy Environ. Sci..

[19] Sun C., Yang P.P., Nan Z., Tian C.B., Cai Y.T., Chen J.F., Qi F.F., Tian H.R., Xie L.Q., Meng L.Y. (2023). Well-Defined Fullerene Bisadducts Enable High-Performance Tin-Based Perovskite Solar Cells. Adv. Mater..

[20] Zhang Z.J., Li D.B., Dong Z.L., Jiang Y., Li X., Chu Y.Q., Xu J.Y. (2023). Lead-Free Cs_2_AgBiBr_6_ Nanocrystals Confined in MCM-48 Mesoporous Molecular Sieve for Efficient Photocatalytic CO_2_ Reduction. Sol. RRL.

[21] Qian J.Y., Hu H., Liang Y., Zhang Z.J. (2024). Mesoporous TiO_2_ matrix embeded with Cs_2_CuBr_4_ perovskite quantum dots as a step-scheme-based photocatalyst for boosting charge separation and CO_2_ photoconversion. Appl. Surf. Sci..

[22] Dong Z.L., Su S.W., Zhang Z.J., Jiang Y., Xu J.Y. (2023). NiFe-Layered Double Hydroxides/Lead-free Cs_2_AgBiBr_6_ Perovskite 2D/2D Heterojunction for Photocatalytic CO_2_ Conversion. Inorg. Chem..

[23] Yin W.-J., Shi T., Yan Y. (2014). Unusual defect physics in CH_3_NH_3_PbI_3_ perovskite solar cell absorber. Appl. Phys. Lett..

[24] Buin A., Pietsch P., Xu J.X., Voznyy O., Ip A.H., Comin R., Sargent E.H. (2014). Materials Processing Routes to Trap-Free Halide Perovskites. Nano Lett..

[25] Yin W.J., Shi T.T., Yan Y.F. (2014). Unique Properties of Halide Perovskites as Possible Origins of the Superior Solar Cell Performance. Adv. Mater..

[26] Chung I., Song J.-H., Im J., Androulakis J., Malliakas C.D., Li H., Freeman A.J., Kenney J.T., Kanatzidis M.G. (2012). CsSnI_3_: Semiconductor or Metal? High Electrical Conductivity and Strong Near-Infrared Photoluminescence from a Single Material. High Hole Mobility and Phase-Transitions. J. Am. Chem. Soc..

[27] Xu P., Chen S.Y., Xiang H.J., Gong X.G., Wei S.H. (2014). Influence of Defects and Synthesis Conditions on the Photovoltaic Performance of Perovskite Semiconductor CsSnl(3). Chem. Mater..

[28] Liu Q., Li A.K., Chu W.B., Prezhdo O.V., Liang W.Z. (2021). Influence of intrinsic defects on the structure and dynamics of the mixed Pb-Sn perovskite: First-principles DFT and NAMD simulations. J. Mater. Chem. A.

[29] Lany S., Zunger A. (2008). Assessment of correction methods for the band-gap problem and for finite-size effects in supercell defect calculations: Case studies for ZnO and GaAs. Phys. Rev. B.

[30] Un-Gi J., Chol-Jun Y., Yun-Hyok K., Yong-Guk C., Wei H., Shuzhou L. (2019). First-Principles Study on Structural, Electronic, and Optical Properties of Inorganic Ge-Based Halide Perovskites. Inorg. Chem..

[31] Qian J.Y., Xu B., Tian W.J. (2016). A comprehensive theoretical study of halide perovskites ABX_3_. Org. Electron..

[32] Yunakova O.N., Miloslavsky V.K., Kovalenko E.N., Kovalenko V.V. (2014). Effect of structural phase transitions on the exciton absorption spectrum of thin CsPbCl_3_ films. Low Temp. Phys..

[33] Stoumpos C.C., Malliakas C.D., Peters J.A., Liu Z., Sebastian M., Im J., Chasapis T.C., Wibowo A.C., Chung D.Y., Freeman A.J. (2013). Crystal Growth of the Perovskite Semiconductor CsPbBr_3_: A New Material for High-Energy Radiation Detection. Cryst. Growth Des..

[34] Chen H., Li M.H., Wang B., Ming S., Su J. (2021). Structure, electronic and optical properties of CsPbX3 halide perovskite: A first-principles study. J. Alloys Compd..

[35] Ahmad W., Khan J., Niu G., Tang J. (2017). Inorganic CsPbI3 Perovskite-Based Solar Cells: A Choice for a Tandem Device. Sol. RRL.

[36] Borriello I., Cantele G., Ninno D. (2008). Ab initio investigation of hybrid organic-inorganic perovskites based on tin halides. Phys. Rev. B.

[37] Sabba D., Mulmudi H.K., Prabhakar R.R., Krishnamoorthy T., Baikie T., Boix P.P., Mhaisalkar S., Mathews N. (2015). Impact of Anionic Br- Substitution on Open Circuit Voltage in Lead Free Perovskite (CsSnI_3-x_Br_x_) Solar Cells. J. Phys. Chem. C.

[38] Lin Z.G., Tang L.C., Chou C.P. (2008). Characterization and properties of infrared NLO crystals: AGeX_3_ (A = Rb, Cs; X = Cl, Br). J. Cryst. Growth.

[39] Tang L.C., Chang C.S., Huang J.Y. (2000). Electronic structure and optical properties of rhombohedral CsGeI3 crystal. J. Phys.-Condens. Matter.

[40] Hao F., Stoumpos C.C., Cao D.H., Chang R.P.H., Kanatzidis M.G. (2014). Lead-free solid-state organic-inorganic halide perovskite solar cells. Nat. Photonics.

[41] Lai M.L., Tay T.Y.S., Sadhanala A., Dutton S.E., Li G.R., Friend R.H., Tan Z.K. (2016). Tunable Near-Infrared Luminescence in Tin Halide Perovskite Devices. J. Phys. Chem. Lett..

[42] Zhong A.G. (2017). Dissecting the nature of halogen bonding interactions from energy decomposition and wavefunction analysis. Monatsh. Chem..

[43] Zhong A.G., Chen D., Li R.R. (2015). Revisiting the beryllium bonding interactions from energetic and wavefunction perspectives. Chem. Phys. Lett..

[44] Ju M.G., Dai J., Ma L., Zeng X.C. (2017). Perovskite Chalcogenides with Optimal Bandgap and Desired Optical Absorption for Photovoltaic Devices. Adv. Energy Mater..

[45] Swift M.W., Lyons J.L. (2021). Deep levels in cesium lead bromide from native defects and hydrogen. J. Mater. Chem. A.

[46] Wei S.H. (2004). Overcoming the doping bottleneck in semiconductors. Comput. Mater. Sci..

[47] Ball J.M., Petrozza A. (2016). Defects in perovskite-halides and their effects in solar cells. Nat. Energy.

[48] Wang Q., Shao Y.C., Xie H.P., Lyu L., Liu X.L., Gao Y.L., Huang J.S. (2014). Qualifying composition dependent p and n self-doping in CH_3_NH_3_PbI_3_. Appl. Phys. Lett..

[49] Maiti A., Chatterjee S., Peedikakkandy L., Pal A.J. (2020). Defects and Their Passivation in Hybrid Halide Perovskites toward Solar Cell Applications. Sol. RRL.

[50] Zhang X., Turiansky M.E., Van de Walle C.G. (2021). All-inorganic halide perovskites as candidates for efficient solar cells. Cell Rep. Phys. Sci..

[51] Zhang X., Turiansky M.E., Shen J.-X., Van de Walle C.G. (2020). Iodine interstitials as a cause of nonradiative recombination in hybrid perovskites. Phys. Rev. B.

